# MicroRNA-105 is involved in TNF-α-related tumor microenvironment enhanced colorectal cancer progression

**DOI:** 10.1038/s41419-017-0048-x

**Published:** 2017-12-13

**Authors:** Zetao Shen, Rui Zhou, Chen Liu, Yaofeng Wang, Wanqi Zhan, Ziyun Shao, Jian Liu, Feifei Zhang, Lijun Xu, Xinying Zhou, Lu Qi, Feng Bo, Yanqing Ding, Liang Zhao

**Affiliations:** 1 Department of Pathology, Nanfang Hospital, Southern Medical University, Guangzhou, China; 2 Department of Pathology, School of Basic Medical Sciences, Southern Medical University, Guangzhou, China; 3 Guangdong Province Key Laboratory of Molecular Tumor Pathology, Southern Medical University, Guangzhou, China; 4 School of Biomedical Sciences, The Chinese University of Hong Kong, Hong Kong, China; 5 Department of Nephrology, Wuhan General Hospital of Guangzhou Military Command of Chinese PLA, Guangzhou, China

## Abstract

TNF-α is a central proinflammatory cytokine contributing to malignant tumor progression in tumor microenvironment. In this study, we found the upregulation of miR-105 in colorectal cancer was associated with aggressive phenotype, and the enhanced expression of miR-105 was required for TNF-α-induced epithelial–mesenchymal transition (EMT). The expression of miR-105 was remarkably stimulated by TNF-α in a time-dependent manner using real-time qPCR analysis. Inhibition of miR-105 remarkably weakened the aggressive effects of TNF-α through preventing the activation of NF-κB signaling and the initiation of EMT. Furthermore, miR-105 was demonstrated directly targeted on the 3′-UTRs of RAP2C, a Rap2 subfamily of small GTP-binding protein. Consistently, suppression of RAP2C stimulated the role of miR-105, which dramatically promoted the invasion and metastasis of CRC cells. Thalidomide, a TNF-α and NF-κB inhibitor, significantly weakened the metastasis and homing capacity of miR-105-overexpressed CRC cells in nude mice. Our investigation initiatively illustrated the modulatory role of miR-105 in TNF-α-induced EMT and further CRC metastasis. We also offer a better understanding of TNFα-induced metastasis and suggest an effective therapeutic strategy against CRC metastasis.

## Introduction

Tumor necrosis factor-alpha (TNF-α), a critical proinflammatory cytokine intensively studied in the immune system, is also established to regulate the process of immunity, cell homeostasis, and tumor progression^1^. As well as other inflammatory mediators brought by the influx of inflammatory cells in the cancer stroma, TNF-α is a vital participant in tumor progression through enhancing cancer cell proliferation, survival, and migration^2,3^. TNF-α significantly promotes tumor lymphangiogenesis, lymphatic metastasis^4^, and cerebral metastasis of breast cancer^5^. TNF-α also function as an important mediator of the colitis-related colorectal carcinogenesis^6^. In several cancer cells, TNF-α produced by both tumor and immune cells contributes to cancer initiation and progression probably through facilitating epithelial–mesenchymal transition (EMT)^7–10^.

Inflammatory microenvironment changes are frequently accompanied by molecular alternations in tumor tissues. MicroRNAs (miRNAs) are considered as potential mediators that regulated their targeting genes through binding to the 3′-untranslated region (UTR) of mRNA transcripts^11^. Hsa-miR-19a was proved to participate in lymph metastasis and mediates TNF-α-induced epithelial–mesenchymal transition in colorectal cancer^12^. Several miRNAs are aberrantly expressed in CRC, and their dysregulation constantly result in cancer progression and clinical outcome^13–16^. Nevertheless, identification of miRNAs involved in proinflammatory factors induced initiation and progression of colorectal cancer, still requires further and extensive investigations.

In this article, our investigation illustrated a novel molecular mechanism underlying the metastatic behavior of colorectal cancer cells. It would undoubtedly prompt the application of miRNA-based technology in therapeutic strategies against CRC metastasis.

## Materials and methods

### Chemicals

Unless otherwise specified, all chemicals were purchased from Sigma-Aldrich (St. Louis, MO, USA), enzymes from New England Biolabs (Ipswich, MA, USA), and culture medium from Gibco Invitrogen (Carlsbad, CA, USA). Lipofectamine 2000 and Trizol reagents were purchased from Invitrogen (Carlsbad, CA, USA). The nucleoprotein and cytoplasm-protein extraction kit was obtained from FD Bioscience (Hangzhou, Zhejiang, China). Recombinant human TNF-α was obtained from Peprotech (Rocky Hill, NJ, USA). The peptide was first diluted in water as stock and further diluted in medium containing bovine serum albumin at a final concentration of 20 μg/ml.

### Antibodies

Antibodies targeting E-cadherin, N-cadherin, β-catenin, fibronectin, vimentin, β-actin, and all unconjugated secondary antibodies were purchased from Santa Cruz Biotechnology (Santa Cruz, CA, USA); Antibodies against ZO-1, p65, p-IKK, IKKβ, p-IKBα, IKBα, and IKKα were obtained from Cell Signaling Technology (Danvers, MA, USA); Antibody to RAP2C was purchased from Abcam (Cambridge, MA, USA); Antibody targeting β-tubulin was obtained from Ray Antibody (Beijing, China) and antibody to Histone H3 (K4) was purchased from Bioworld (St. Louis Park, MN, USA). All the primary antibodies were used at a dilution of 1:1000 in PBST Buffer (137 mM NaCl, 2.7 mM KCl, 8 mM Na_2_HPO_4_, 1.46 mM KH_2_PO_4_, 0.05% Tween-20) with 5% non-fat dry milk.

### Cell culture

The normal human colon epithelial cell line FHC and CRC cell lines including LS174T, LoVo, HT29, SW620, SW480, and HCT116 were purchased from the Cell Bank of the Chinese Academy of Sciences (Shanghai, China). All cells were cultured following instructions in RPMI supplemented with 10% fetal bovine serum (FBS) at 37 °C with a humidity of 90–95% and 5% CO_2_^17^.

### Tumor tissue samples

Fresh primary CRC specimens with paired normal colorectal tissues were obtained from the Tumor Tissue Bank of Nanfang Hospital. Pathological diagnosis was made in the department of pathology before patients undergoing elective surgery in Nanfang Hospital between 2007 and 2010. All experiments performed are endorsed by the Ethics Committee of Southern Medical University and complied with the Declaration of Helsinki. No informed consent was required because data were going to be analyzed anonymously.

### Micro-RNA and interference RNA transfection

miR-105-5p mimic, nonspecific miR control, anti-miR-105-5p, and a nonspecific anti-miR control were all purchased from GenePharma (Shanghai, China). They were transfected at a working concentration of 100 nM using Lipofectamine 2000 reagent. RNA samples were extracted from subconfluent cells in the exponential phase of growth.

### Cell proliferation assays

Cell proliferation assays were carried out using Cell Counting Kit 8 (CCK8) (Dojindo; Kumamoto, Japan). Cells were plated in 96-well plates at a density of 1 × 10^4^ cells per well and cultured in the growth medium. At the indicated time points, the number of cell in triplicate wells was measured using the absorbance at 450 nm of reduced WST-8 (2-(2-methoxy-4-nitrophenyl)-3-(4-nitrophenyl)-5-(2,4-disulfo-phenyl)-2H-tetrazolium,monosodium salt).

### RNA isolation, reverse transcription, and real-time quantitative PCR

Total RNA was extracted using Trizol reagent according to manufacturer’s protocol and our previous report^15^. To quantitate the expression of miR-105, total RNA was polyadenylated and underwent Reverse transcription using an ALL-in-One^TM^ miRNA qRT-PCR Detection System (GeneCopoeia). Reverse transcription of RAP2C was performed as previously described^15^. Real-time quantitative PCR (qPCR) was carried out using SYBR Green PCR master mix (Applied Biosystems; Foster City, CA, USA) on ABI 7500HT system. GAPDH or U6 snRNA was chosen to be endogenous control. All the primers were purchased from Invitrogen (Carlsbad, CA, USA). The expression level of each targeted gene was normalized as fold change compared with control or reference group. Fold changes were calculated through relative quantification ($2^{ - \Delta \Delta {\mathrm{C}}T}$).

### Wound-healing and Transwell migration assay

HCT116 and SW480 CRC Cells were seeded onto 24-well plates and grown to confluence. After starvation in serum-free medium for 12 h, cells were gently wounded using pipette tips. Images were taken at 0, 12, and 24 h after wounding. The motility of CRC cells was assessed through the distance between the wound edges. CRC cells cultured in 200 μl 1640 were seeded in the upper chamber of the Transwell insert, while 600 μl medium with 20% FBS was put in the lower chamber. Cells were cultured for 24 h and that invaded to the underside of the membrane were fixed and stained with crystal violet. Cells were counted from five random fields of view and the mean value was calculated from three independent experiments.

### Animals

All animal experiments were carried out with the approval of the Southern Medical University Animal Care and Use Committee in accordance with the guidelines for the ethical treatment of animals. Nude *nu/nu* mice were maintained in a barrier facility in racks filtered with a high-efficiency particulate air filter. The animals were fed an autoclaved laboratory rodent diet. The mice in this study were purchased from the Experimental Animal Centre of Southern Medical University, which is certified by the Guangdong Provincial Bureau of Science.

### Lung colonization assay

HCT116 CRC cells were transduced with LV-miR-NC and LV-miR-105 Lentivirus for 72 h. CRC cells (5 × 10^6^ per mouse) w/o miR-105 overexpression were injected into the tail veins of 6-week-old nude mice, respectively. Thalidomide (1.5 mg/kg) was intraperitoneally injected everyday to control and miR-105 stable overexpressing mice to observe the influence of TNF-α inhibitor on miR-105-induced tumor metastasis. The mice were all killed 8 weeks later, at which time individual organs were removed and metastatic tissue was analyzed using hematoxylin and eosin (H&E). Number of metastatic lung nodules in individual mice was counted under the microscope.

### Bioinformatics

Potential miRNA targets were predicted using three publicly available algorithms: TargetScan, Diana, and miRDB^18^. The predicted targets were chosen only when they were positive in all three analyses. Normalized expression values for mRNA and miRNA data in colorectal cancer were downloaded from The Cancer Genome Atlas (TCGA, http://cancergenome.nih.gov/) and 358 samples were used. All analyses were performed with R (R is a language and environment for statistical computing).

### miRNA target validation

The full-length of RAP2C 3′UTR were amplified by PCR and cloned at the downstream of the firefly luciferase gene in the psiCHECK-2 vector (Promega; Madison, WI, USA). This vector was named wild-type (wt) 3′UTR. Site-directed mutagenesis of the miR-105-binding site in the RAP2C 3′UTR was carried out using the GeneTailor Site-Directed Mutagenesis System (Invitrogen) and named mutant (mt) 3′UTR. For reporter assays, the wt or mt 3′UTR vector and miR-105 mimic or inhibitor were co-transfected. Luciferase activity was measured at 48 h after transfection using Dual-Luciferase Reporter Assay System (Promega, Madison, WI, USA).

### Preparation of lentiviral vectors

A DNA fragment corresponding to pre-miR-105 and the flanking sequence was amplified from human genomic DNA and then cloned into pGLV3/H1/GFP + puro lentiviral vector (http://www.genepharma.com). The production, purification, and titration of lentivirus were performed as previously described^16^. The packaged lentiviruses were named LV-miR-105. The empty lentiviral vector LV-con was used as a control.

### Statistical analysis

Data were analyzed using SPSS version 19.0 statistical software (SPSS, Chicago, USA). For qRT-PCR assay, all values were expressed as the mean ± SD and the data were statistically analyzed by *T* test to assess the statistical significance of the differences. *P* values are labeled in the figures.

## Results

### Overexpression of TNF-α is associated with colorectal cancer progression

**Fig. 1:**
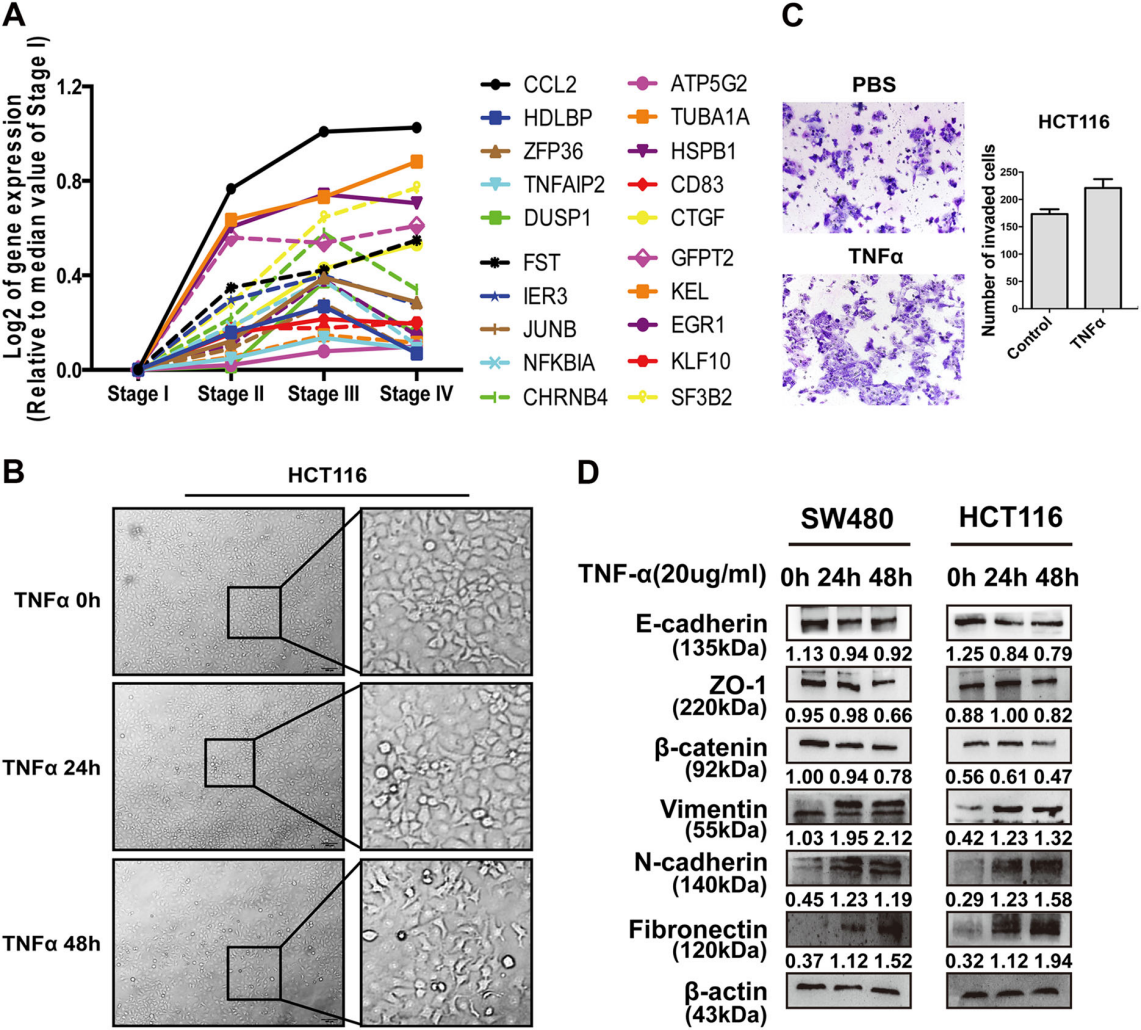
Overexpression of TNF-α is associated with colorectal cancer progression **a** Line chart showing the relationship between TNF-α-NF-κB targeted genes and CRC progression. Twenty representative genes were chosen for line chart plotting. **b** The morphology of cultured HCT116 cells in response to TNF-α for different periods of time (0–48 h) was observed under an inverted microscope. **c** Transwell assay on PBS and recombinant human TNF-α treated SW480 cells. The cells were counted under a microscope in five randomly selected fields. Bars of below represent the number of invaded cells (mean ± SD, *n* = 3). **d** Western blotting analysis was performed to detect EMT markers in both SW480 and HCT116 cells after TNF-α treatment for 24 h and 48 h (**a** representative set of data was shown, *n* = 5)

TNF-α was demonstrated more abundantly expressed in CRC tissues compared with adjacent normal mucosa, and its expression is positively correlated with Dukes’ stages^19,20^. Normalized expression values of mRNA data in colorectal cancer downloaded from The Cancer Genome Atlas and a published high-throughput microarray dataset (NCBI/GEO/GSE39582; *n* = 566) were used for line chart plotting. Only 20 representative genes and the median value of their expression in different colorectal cancer stages were shown in the line chart. We identified a correlation between the transcription of TNF-α/ NF-κB-induced genes and CRC progression (Fig. 1a). TNF-α/ NF-κB-induced genes was selected based on published papers^21,22^. In addition, TNF-α obviously switched HCT116 cells from a fibroblastic-like appearance to a cobblestone-like phenotype after 48 h of recombinant human TNF-α treatment, indicating an epithelia-mesenchymal transition (Fig. 1b). Coupled with the morphologic changes of EMT, TNF-α could also enhance the invasive ability of HCT116 cells in a time-dependent manner (Fig. 1c). Both SW480 and HCT116 cells were used to elucidate the effects of TNF-α on EMT markers and only a representative set of data was displayed. Recombinant TNF-α significantly decreased the expression of epithelial markers including E-cadherin, β-catenim, and ZO-1, whereas increased that of mesenchymal markers containing vimentin, N-cadherin, and fibronectin (Fig. 1d). In order to make those changes easily to be traced, gray values generated by Image J were displayed on the blots.

### miR-105 is involved in TNF-α related colorectal cancer progression

**Fig. 2:**
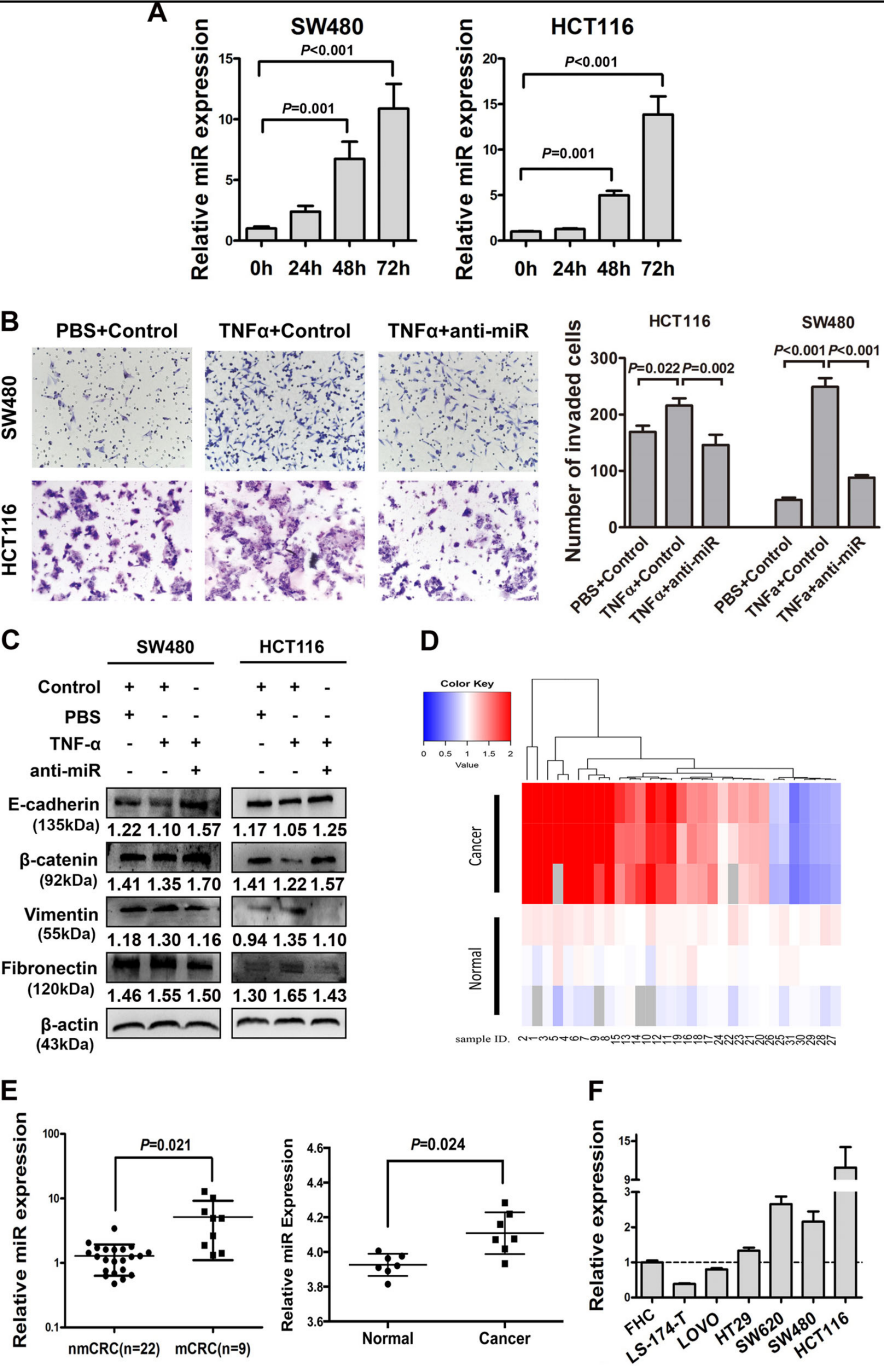
miR-105 is involved in TNF-α related colorectal cancer progression **a** Real-time PCR analysis of miR-105 expression in TNF-α treated SW480 and HCT116 cells for different time periods (0–72 h). The values represented relative miR-105 level after normalization to the expression of U6 (mean ± SD, *n* = 3). **b** Transwell assay on the effect of anti-miR-105 in TNFα treated HCT116 cells. Bars of the right panel represent the number of invaded cells (mean ± SD, *n* = 3). **c** Western blotting analysis was performed to detect EMT markers in both SW480 and HCT116 cells after TNF-α treatment for 24 h and 48 h. The effects of anti-miR-105 in TNFα treated HCT116 cells were also studied (**a** representative set of data was shown, *n* = 4). **d** Heat map depicting the differential expression of miR-105 in 31 paired human CRC tissues and their adjacent normal mucosa tissues. Red and blue indicate high and low miR-105 expression. **e** The expression of miR-105 in CRC tissues with or without metastasis. nmCRC denotes CRC tissues without metastasis. mCRC denotes CRC tissues with lymph node metastasis. The right panel shows the aberrantly expression of miR-105 in human CRC tissues and their adjacent normal mucosa analysis using a published high-throughput microarray dataset (NCBI/GEO/GSE68377). **f** Expression of miR-105 in six CRC cell lines compared with the normal human colon epithelial cell line FHC (mean ± SD, *n* = 3)

Inflammatory microenvironment changes are frequently accompanied by molecular alternations in tumor tissues and miRNAs are usually considered as potential mediators. In this study, we found the expression of miR-105 was remarkably stimulated by TNF-α in a time-dependent manner using real-time qPCR analysis (*P* < 0.05, Fig. 2a). TNF-α enhanced cell migration could be totally abrogated by silencing of miR-105 in HCT116 cells (Fig. 2b). Furthermore, introduction of anti-miR-105 in TNF-α-treated cells remarkably rescued the suppression of epithelial markers and restrained the stimulation of mesenchymal markers, suggesting an essential role for miR-105 in TNF-α-inducing EMT (Fig. 2c).

### miR-105 is overexpressed in CRC tissues and cell lines

Real-time qPCR was used to measure the expression of miR-105 transcripts in CRC tissues and cell lines. Thirty-one pairs of fresh CRC tissues with adjacent normal mucosa were measured, among which nine cases were metastatic CRC (mCRC) while 22 cases were non-metastatic CRC (nmCRC). Heat map clearly showing the expression of miR-105 in both CRC tissues and their adjacent normal mucosa (Fig. 2d). Our data indicated that miR-105 was significantly upregulated in 77.4% (24/31) of CRC tissues examined compared to the adjacent normal tissues (*P* = 0.031). Moreover, the expression of miR-105 in mCRC tissues was dramatically higher than that in nmCRC tissues (*P* = 0.021, Fig. 2e, left panel). Aberrantly expressed miRNAs were also investigated through analyzing a published high-throughput microarray dataset (NCBI/GEO/GSE68377; *n* = 7). Consistently, the microarray result showed that the expression of miR-105 was upregulated an average of 1.046 fold in CRC tumor tissues compared with normal colorectal mucosa tissue specimens (*P* = 0.024, Fig. 2e, right panel). Increased expression of miR-105 was also found in CRC cell lines including HT29, SW620, SW480, and HCT116, compared with normal human colon epithelial cell line FHC (Fig. 2f).

### Overexpression of miR-105 enhances CRC cell migration and epithelial–mesenchymal transition in vitro

**Fig. 3:**
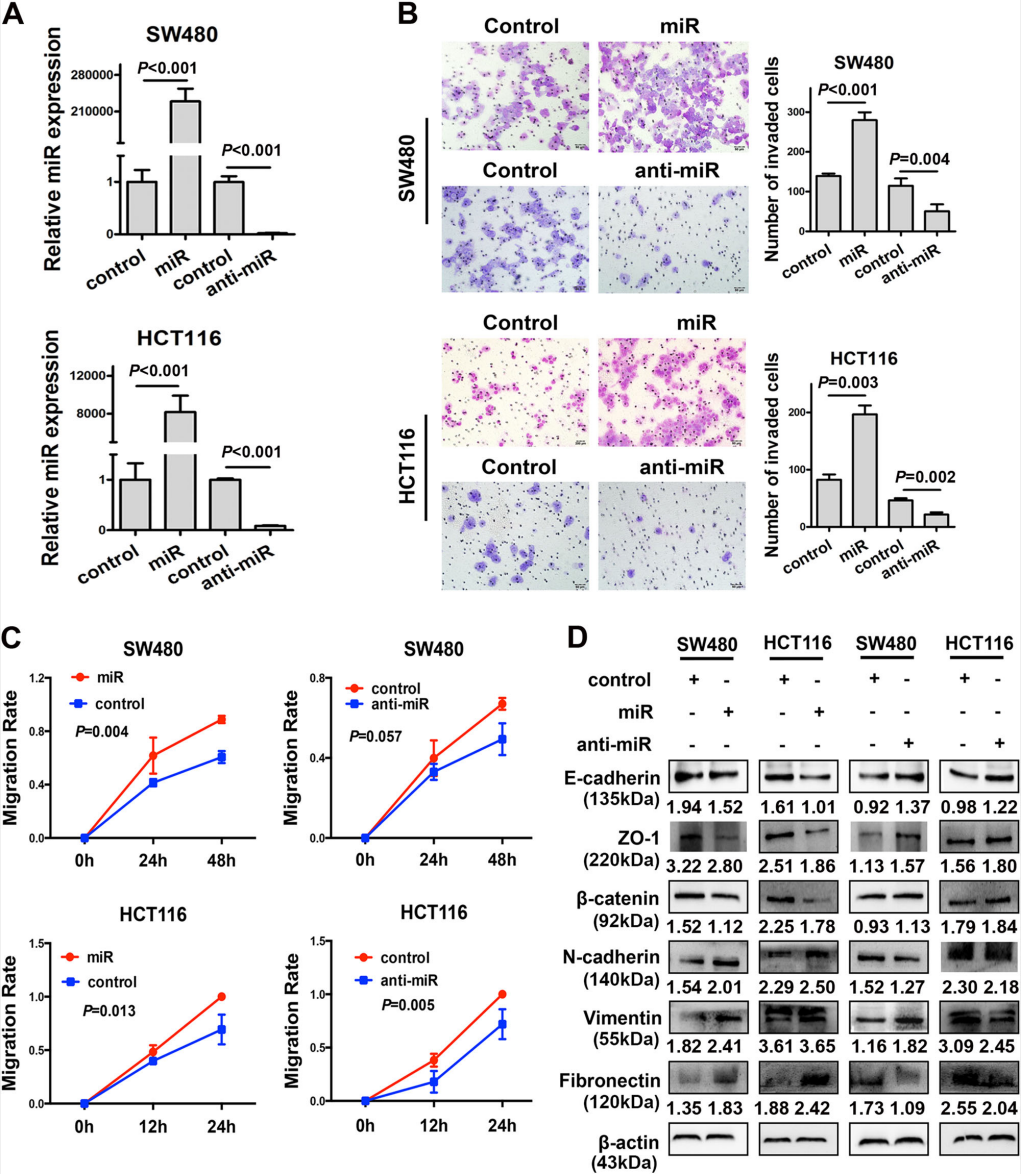
Overexpression of miR-105 promoted aggressive phenotypes of CRC cells *in vitro* **a** Real-time PCR analysis of miR-105 expression in control, miR-105, and anti-miR-105-transfected cells. The values represented relative miR-105 level after normalization to the expression of U6 (mean ± SD, *n* = 3). **b** Transwell assay on control, miR-105, and anti-miR-105-transfected SW480 and HCT116 cells. The cells were counted under a microscope in five randomly selected fields. Bars of the right panel represent the number of invaded cells (mean ± SD, *n* = 3). **c** Migration index of wound-healing assay on control, miR-105, and anti-miR-105 overexpressed SW480 and HCT116 cells. The distance migrated by treated cells was relative to that migrated by control cells (mean ± SD, *n* = 3). **d** Expression of the epithelial markers and the mesenchymal markers in control, miR-105, or anti-miR-105-transfected SW480 and HCT116 cells were assessed by western blot. β-actin was used as a loading control (**a** representative set of data was shown, *n* = 3)

To evaluate the effects of miR-105 on the malignant behavior of colorectal cancer cells, we transfected HCT116 and SW480 cells with miR-105 mimic oligonucleotides. Anti-miR-105 oligonucleotides were used to silence the expression of miR-105. Transfection efficiency was monitored through detecting the expression level of miR-105 using real-time qPCR (*P < *0.05, Fig. 3a). Transwell and wound-healing assays indicated that overexpression of miR-105 dramatically increased the migration and motility potential of CRC cells (*P < *0.05, Fig. 3b, c and Supplementary Fig. 1A, C). In contrast, cells transfected with anti-miR-105 oligonucleotides significantly reduced the aggressive effects of CRC cells (*P < *0.05, Fig. 3b, c and Supplementary Fig. 1B, D). CCK-8 assay was also performed to reveal the influence of miR-105 on cell proliferation. However, overexpression of miR-105 did not influence the proliferation of either HCT116 or SW480 cells (Data not shown). We performed western blot assays to investigate effects of exogenous miR-105 on the expression of EMT makers in both HCT116 and SW480 cells. Our data supported a positive role of miR-105 on the process of EMT since it dramatically suppressed the expression of epithelial markers (E-cadherin, β-catenin, and ZO-1), whereas increased that of mesenchymal markers (N-cadherin, fibronectin, and vimentin). In contrast, suppression of miR-105 remarkably inhibited EMT process in CRC cells indicated by the increased expression of epithelial markers and suppressed expression of mesenchymal markers (Fig. 3d). These findings supported a stimulatory effect of miR-105 on EMT of CRC cells.

### miR-105 modulates TNF-α-induced epithelial–mesenchymal transition in a NF-κB-dependent pathway in CRC cells

**Fig. 4:**
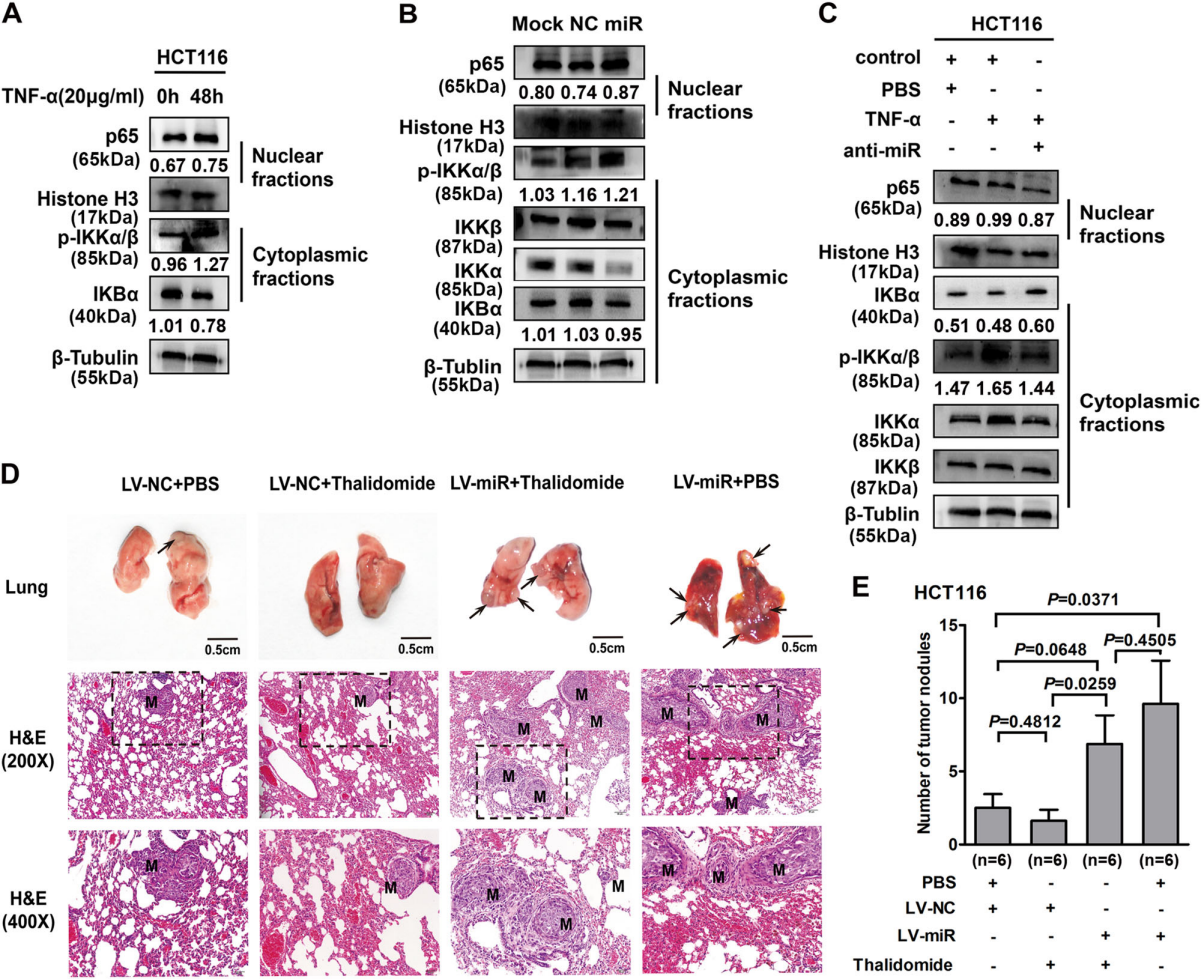
miR-105-mediated TNF-α-induced EMT in a NF-κB-dependent manner in CRC cells **a** Western blotting analysis detected the expression of NF-κB family members in HCT116 cells after TNF-α treatment for 48 h (**a** representative set of data was shown, *n* = 4). **b** Effects of overexpressed miR-105 on the expression of NF-κB family members were detected by western blotting analysis (**a** representative set of data was shown, *n* = 4). **c** Effects of miR-105 on TNF-α treated expression of NF-κB family members (**a** representative set of data was shown, *n* = 4). **d** Tumor cells were injected into nude mice through the tail vein to evaluate the lung homing potential of cells. Thalidomide (1.5 mg/kg) was intraperitoneally injected everyday to observe the influence of TNF-α inhibitor on miR-105-induced tumor metastasis. The size and number of metastatic lung nodules in individual mice was counted under the microscope. The magnification areas indicated metastatic nodes in the lung under H&E staining (**a** representative set of data was shown, *n* = 2). **e** The influence of TNF-α inhibitor on miR-105-induced tumor metastasis. Bars represent the number of metastatic lung nodules

NF-κB pathway is the classic pathway of TNF-α stimulation. Western blot assay demonstrated that NF-κB pathway was activated in response to TNF-α stimulation. Increased expression of cytoplasmic p-IKKα/β and nuclear p65, as well as decreased expression of IKBα in the cytoplasm were detected after 48 h of recombinant TNF-α treatment (Fig. 4a). We also transfected HCT116 cells with miR-105 mimic to detect the effect of miR-105 on NF-κB pathway. The expression level of NF-κB pathway members was detected afterwards. As shown in Fig. 4b, introduction of miR-105 remarkably stimulated the expression of p65 in the nuclear as well as the phosphorylation of IKKα/β and IKBα in the cytoplasm. In contrast, the expression of IKBα, IKKβ, and IKKα decreased in the cytoplasm in response to the overexpression of miR-105 (Fig. 4b). Furthermore, The effects of TNF-α were abolished to a large extent by the transfection of anti-miR-105 through rescuing the expression changes of p65 and p-IKKα/β (Fig. 4c).

### Inhibition of TNF-α/NF-κB pathway attenuates the invasive and metastatic potential of miR-105-overexpressing CRC cells in vivo

To investigate the biological effect of miR-105 in CRC progression in vivo, we used a lentivirus-based system to establish miR-105 stable overexpressing CRC cell lines. The expression of miR-105 was remarkably increased in both LV-miR-105 infected SW480 and HCT116 cells compared with LV-miR-NC infected cells (*P* < 0.05, Supplementary Fig. 2A). Moreover, miR-105 stable overexpressing SW480 and HCT116 cells obviously enhanced migration and motility capacity (*P* < 0.05, Supplementary Fig. 2B, C), as well as the process of EMT (Supplementary Fig. 2D).

We injected miR-105 stable overexpressing HCT116 cells into the tail vein of nude mice to observe its effect on the potential of homing capacity. More and larger tumor nodules were formed in the lung of miR-105-overexpressing group compared with control group. Moreover, we investigated that high level of miR-105 expression tends to shorter overall survival of the animals (Data not shown). Thalidomide (1.5 mg/kg), a TNF-α and NF-κB inhibitor, was intraperitoneally injected everyday to nude mice that injected miR-105 stable overexpressing HCT116 cells in the tail vain. TNF-α inhibitor not only suppress the metastasis and homing capacity of HCT116 cells but also weakened that of miR-105-overexpressed HCT116 cells through forming less and smaller tumor nodules (Fig. 4d, e). The in vivo data further support our notion that miR-105 is a pivotal modulator of TNF-α-induced CRC progression.

### RAP2C is the direct target of miR-105

**Fig. 5:**
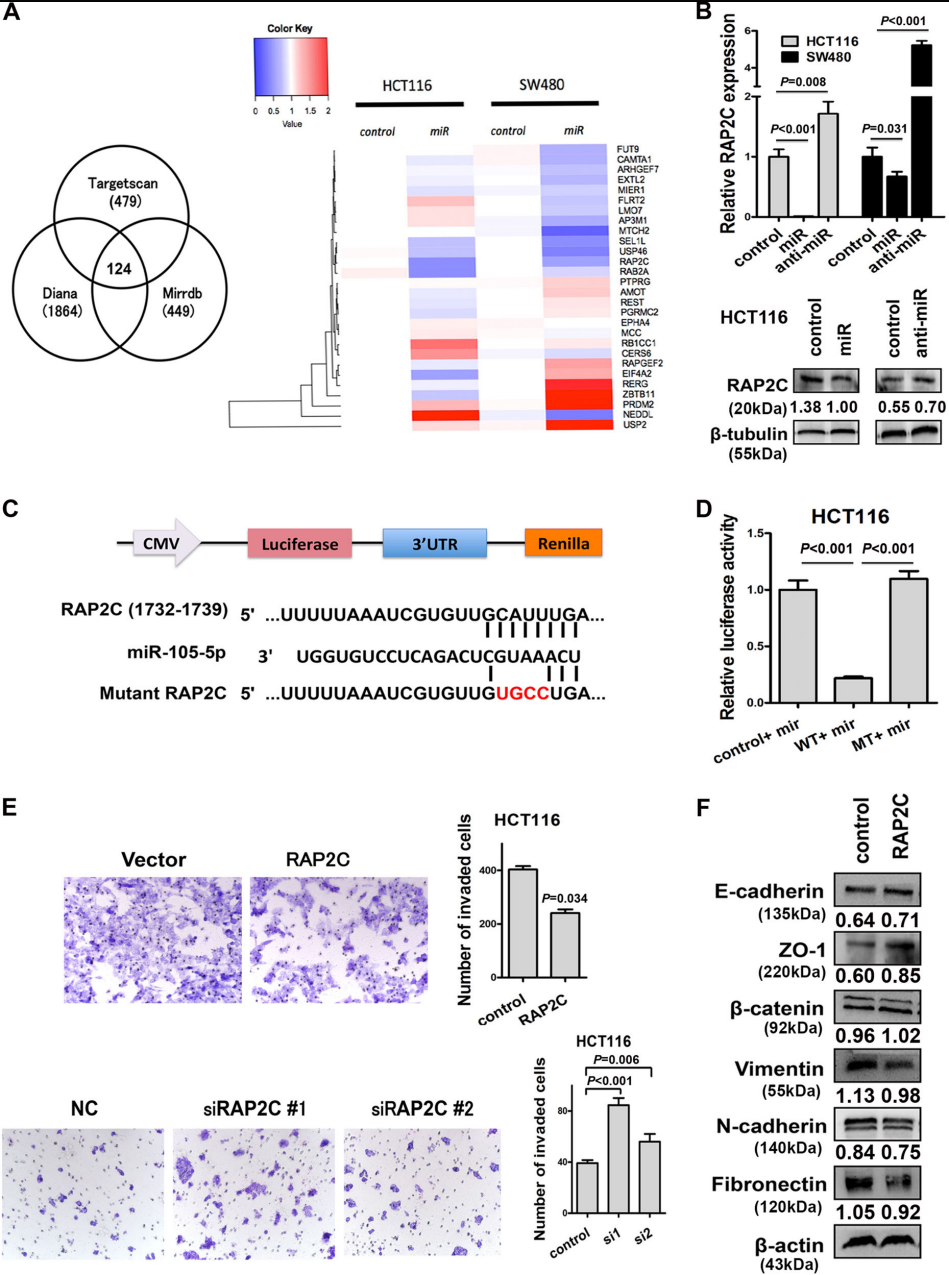
RAP2C is the direct target of miR-105 in CRC progression **a** Left panel: The Venn diagram indicated the 124 genes were overlapped in three publicly available bioinformatics algorithms (TargetScan, PicTar, miRanda). Twenty-eight genes were selected to verify their potential roles on tumor metastasis. Right panel: Heat map indicated the expression of 28 selected genes in miR-105 overexpressed SW480 and HCT116 cells. **b** Upper is the real-time PCR analysis of RAP2C expression in control, miR-105, and anti-miR-105-transfected CRC cells (mean ± SD, *n* = 3). Below is the western blotting analysis of RAP2C proteins in HCT116 cells transfected with miR-105 mimic and anti-miR (**a** representative set of data was shown, *n* = 3). **c** Predicted miR-105 target sequence in RAP2C 3′UTR and mutant containing 4 mutated nucleotides in the seed sequence of miR-105 (mt). **d** Luciferase reporter assays were performed in 293T cells, with co-transfection of wt or mt 3′UTR and miR-105 mimic, as indicated (mean ± SD, *n* = 3). **e** Transwell assay on RAP2C overexpressing or silencing HCT116 cell. Bars of the right panel represent the number of invaded cells (mean ± SD, *n* = 3). **f** Effects of RAP2C on the expression EMT markers

Three publicly available bioinformatics algorithms (Targetscan, Diana, and miRDB) were adopted to search target genes of miR-105. Total 124 genes were selected eligible for all three bioinformatics algorithms and 28 genes of them exhibit anti-tumor potential but have not been reported in colorectal cancer yet (Fig. 5a, left panel). We validated the expression of these 28 genes in miR-105-overexpressed HCT116 and SW480 cells using real-time qPCR. Heat map showed that the expression of RAP2C was dramatically decreased in both cell lines (Fig. 5a, right panel). Consistently, silencing miR-105 led to an increased mRNA level of RAP2C (Fig. 5b, upper panel). Immunoblot assay also confirmed that lower expression of RAP2C is correlated with overexpressed miR-105, whereas suppressing miR-105 would enhance RAP2C expression (Fig. 5b, lower panel). Luciferase reporter assay was performed to determine whether miR-105 could directly target the 3′UTR region of RAP2C (WT) that containing miR-105-binding site. A mutation in the putative-binding site in the RAP2C 3′UTR region (MT) was designed (Fig. 5c). The 3′UTR regions of both WT and MT fragments were cloned into luciferase report vectors and co-transfected with miR-105 mimic into HCT116 cells. Significant decrease of luciferase activity was detected in WT vectors after transfected with miR-105 mimic, while the mutation in the putative-binding site abrogated the suppressive effect mediated by miR-105 mimic, thereby confirm a direct interaction between miR-105 and 3′UTR region of RAP2C (Fig. 5d). Undoubtedly, all the facts above supported our assumption that RAP2C is the direct target of miR-105.

### RAP2C suppresses CRC cell migration and epithelial–mesenchymal transition in vitro

We perform transwell assay to study the role of RAP2C on migration and invasion capacity of CRC cells. Indeed, overexpression of RAP2C dramatically weakened the migration and invasion capacity of HCT116 cells, while knockdown of RAP2C could restore its ability on migration and invasion (Fig. 5e). In contrast to the stimulatory effect of miR-105 on EMT, immunoblot assay resulting in a gain of epithelial markers (E-cadherin, ZO-1, and β-catenin) and a losing of mesenchymal markers (vimentin, N-cadherin, and fibronectin) in RAP2C-overexpressed HCT116 cells (Fig. 5f). These results indicated that RAP2C weaken the migration and invasion ability of CRC cells through promoting MET.

### RAP2C plays crucial roles in TNF-α/miR-105/NF-κB-induced EMT of CRC cells

**Fig. 6:**
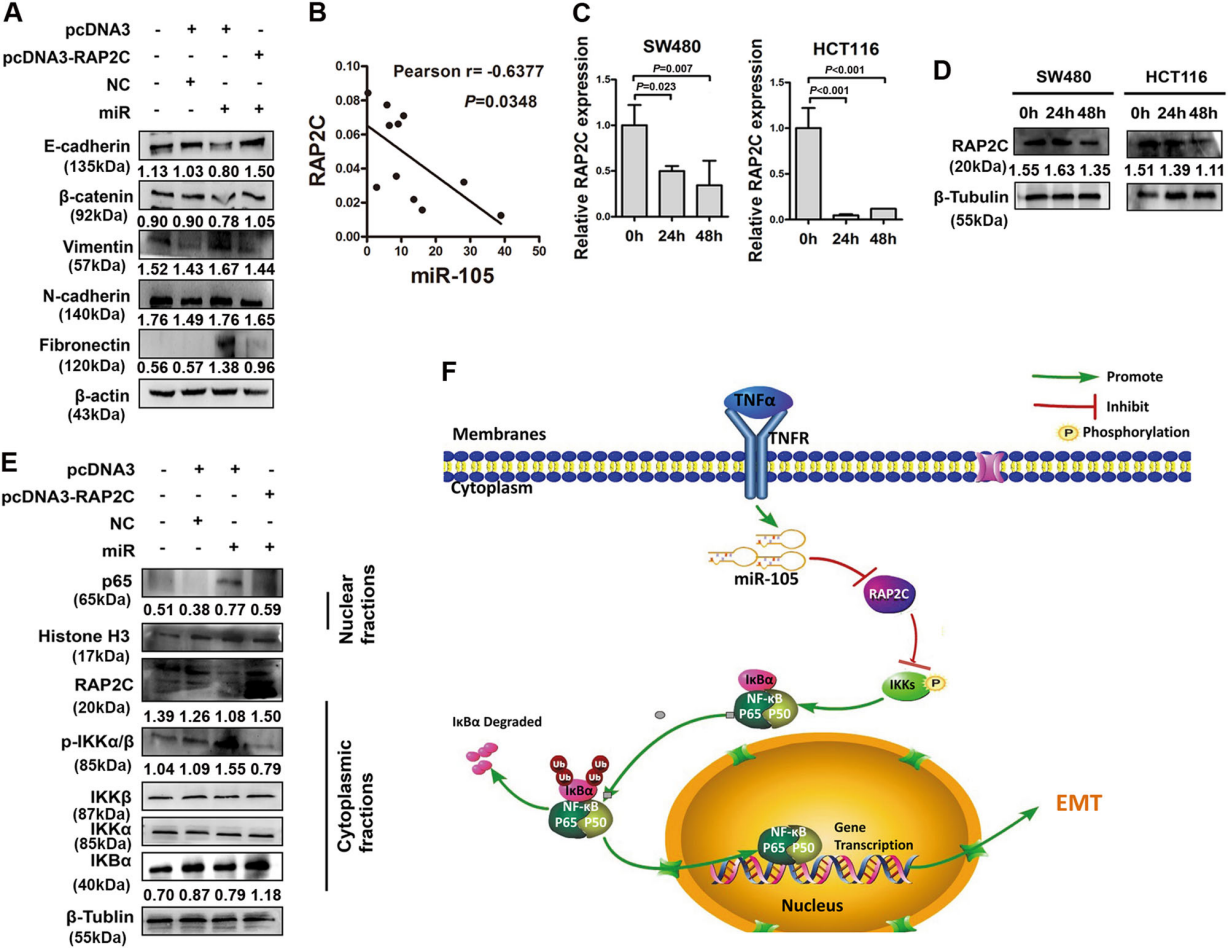
RAP2C plays crucial roles in TNF-α/miR-105/NF-κB-induced EMT of CRC cells **a** Effects of RAP2C on the expression EMT markers in miR-105 overexpressed CRC cells (**a** representative set of data was shown, *n* = 3). **b** The negative correlation between the expression of miR-105 and RAP2C. Expression data of fresh CRC tissues and their adjacent normal mucosa was used for graph drawing. **c** Real-time qPCR analysis of RAP2C expression in response to TNF-α (mean ± SD, *n* = 3). **d** Western blotting analysis detected the expression of RAP2C in response to TNF-α (**a** representative set of data was shown, *n* = 3). **e** Western blotting analysis detected the effects of RAP2C on miR-105 treated expression of NF-κB family members (mean ± SD, *n* = 3). **f** Hypothetic model illustrating that miR-105 modulate the constitutive activation of TNF-α in tumor microenvironment through a NF-κB-dependent pathway to increased CRC development and progression

To evaluate the biological role of RAP2C induced by miR-105, we rescued the expression of RAP2C in miR-105-overexpressed CRC cells through transfection of RAP2C ORF constructs without 3′UTRs. The results showed that exogenous introduction of RAP2C could reverse the impact of miR-105 on EMT status (Fig. 6a). Furthermore, a negative correlation between the expression of miR-105 and RAP2C were detected when we measure their expression level in fresh CRC tissues (Fig. 6b). We detected the expression of RAP2C in response to TNF-α stimulation to analyze the role of RAP2C in TNF-α signaling. Both mRNA and protein expression levels of RAP2C were decreased in HCT116 and SW480 cells in response to TNF-α at 48 h of treatment (Fig. 6c, d). Immunoblot assays indicated that transfection of RAP2C construct obviously suppressed NF-κB pathway in HCT116 cells, nevertheless, silencing of RAP2C could activated that pathway (Supplementary Fig. 3). Introduction of RAP2C without 3′UTR in miR-105-overexpressed HCT116 cells could abrogate the activation of NF-κB pathway (Fig. 6e). Overall, RAP2C function as an important effector of miR-105, involving in the activation of NF-κB pathway to stimulate EMT of CRC cells.

## Discussion

Constitutive secretion of TNF-α from tumor microenvironment is a characteristic of many malignant tumors and its aberrant expression is often associated with poor prognosis. Besides macrophage from tumor microenvironment, TNF-α could also be produced in tumor cells including like B-cell lymphoma and breast and colon carcinoma. Both macrophage and tumor cell secreted TNF-α could be regulated in an autocrine manner to accelerate tumor progression accordingly^7^. Concentration of serum TNF-α in CRC patients is clinically relevant to CRC progression and mRNA transcripts of TNF-α are more abundant in CRC tissues compared with adjacent normal mucous^23,24^. In our study, line chart plotted by 20 representative genes indicated a significant correlation between the expressions of TNF-α/NF-κB-induced genes and CRC progression. This definitely strengthened the tight connection between TNF-α activation and CRC metastasis.

TNF-α produced by both tumor and immune cells from cancer stroma contributed to cancer initiation and progression through facilitating EMT in many malignant tumors^8,9,25^. NF-κB mediated the effects of TNF-α through a snail-dependent or ZEB1/ZEB2-dependent mechanism to suppress E-cadherin and stimulate the following EMT^25^. TNF-α was also reported significantly promoting the process of EMT in colorectal cancer^12,26^. Our results also showed that TNF-α could remarkably facilitate CRC cells switching from a spinal-shaped and fibroblastic-like appearance to a cobblestone-like phenotype.

Although growing evidences emphasized on the importance of miR-105 in cancers, none of previous studies systematically investigated its role in TNF-α-induced metastasis in human CRC. Interestingly, we found the expression of miR-105 is dramatically induced by TNF-α in a dose-dependent manner in CRC cells. Silencing of miR-105 remarkably rescued the suppression of epithelial markers and stimulation of mesenchymal markers brought by TNF-α. TNF-α-enhanced migration ability of CRC cells could also be abrogated by the knockdown of miR-105. NF-κB is extensively studied in TNF-α-induced tumor initiation and progression^25^. As a heterodimeric transcription activator, NF-κB consists of a DNA-binding subunit p50 and a transactivation subunit p65. The activation of NF-κB will translocate itself into the nucleus and transcriptionally activate target genes. Activation of NF-κB is critical for tumor metastasis in many cancer types including CRC^26–29^. Key molecules of NF-κB pathway were studied by western blot assays. Activation of NF-κB pathway was confirmed by the increased expression of cytoplasmic p-IKKα/β and nuclear p65, as well as the decreased expression of IKBα in the cytoplasm in response to TNF-α. Surprisingly, the introduction of anti-miR-105 totally abolished the TNF-α-induced alternation of NF-κB pathway members indicating a modulatory role of miR-105 is through a NF-κB-dependent pathway.

Lung colonization assay was conducted on nude mice to study the homing capacity of CRC cells. We attempt to introduce thalidomide, an inhibitor of both TNF-α^30^ and NF-κB^31^, intraperitoneally injected to nude mice. Surprisingly, thalidomide led to less and smaller tumor nodules in the lung compared with the PBS control group, although without statistical significance because of the limited numbers of nude mice. Moreover, thalidomide tends to generate less and smaller tumor nodules in the lung compared with that injected miR-105 stable overexpressing CRC cells only. Our tentative experiments indicated that TNF-α inhibitor had an anti-tumor effect against colorectal cancer, although further studies still need to be carried out. Evidences above concluded that miR-105 is the downstream modulator of TNF-α/NF-κB-induced CRC metastasis.

As the expression of RAP2C was dramatically downregulated in both HCT116 and SW480 cells compared with the other downregulated genes in response to miR-105, RAP2C was chosen as the main target of miR-105. All the evidences indicated that RAP2C is a direct target of miR-105. RAP2C is a novel member of the Ras family that belongs to small GTPase^32,33^. It contains an ORF of 552bp, encoding a protein of 183 amino acids. RAP2C mainly localizes in the cytoplasm and plasma membrane of cells and its silencing usually cause disturbance of cell junction^34^. RAP2C might act as a positive regulator in SRE-mediated transcriptional regulation through MEK/ ERK pathway. Except rather limited studies on cell junction, no paper was published on the role of RAP2C in tumorgenesis and progression. We hypothesized and initiatively proved the relationship between RAP2C and metastasis of CRC. As expected, the suppressive effects of RAP2C were detected on tumor progression. RAP2C significantly suppressed the EMT-mediated cell migration and metastasis in a NF-κB-dependent pathway in HCT116 cells. Although no direct evidence indicated the interaction between RAP2C directly and NF-κB members, numerous publications illustrated the direct interaction between small GTPase family members and NF-κB pathway. RhoA GTPase could activate Rho-associated Kinase (ROCK) to phosphorylate IKKβ in response to transforming growth factor (TGF)-β1^35^. Rac1 involved in NF-κB activation and IL-8/CXCL8 expression through a Rac1-dependent PI3K/Akt pathway in lung epithelial cells^36^. As a result, RAP2C might work directly on NF-κB by phosphorylation or indirectly through influencing Ras–Raf–MEK–ERK pathway to participate in the process of EMT as well as many fundamental cellular processes including proliferation, survival, and differentiation. Related studies still need to be carried out.

In summary, our studies investigated a novel modulatory role of miR-105 in TNF-α-induced CRC metastasis. We found the overexpression of miR-105 was associated with aggressive phenotype and miR-105 could directly target on RAP2C in a NF-κB-dependent manner to stimulate the migration and invasion of CRC cells through EMT. Hypothetic model illustrating the modulatory role of miR-105 in TNF-α in tumor microenvironment was demonstrated in Fig. 6f. Moreover, our introduction of anti-inflammatory drugs in animal experiments will definitely provide a new direction to make therapeutic improvement in CRC.

## Electronic supplementary material

Supplementary Figures and Tables
