# Supplementary material for: MicroRNA-105 is involved in TNF-α-related tumor microenvironment enhanced colorectal cancer progression
Source: Cell Death Dis. 2017 Dec 13;8(12):3213. doi: 10.1038/s41419-017-0048-x (PMC5870598; doi:10.1038/s41419-017-0048-x)
Supplement: Supplementary file 1 — Supplementary Figures and Tables [file 41419_2017_48_MOESM1_ESM.pdf]

Supplementary Figures

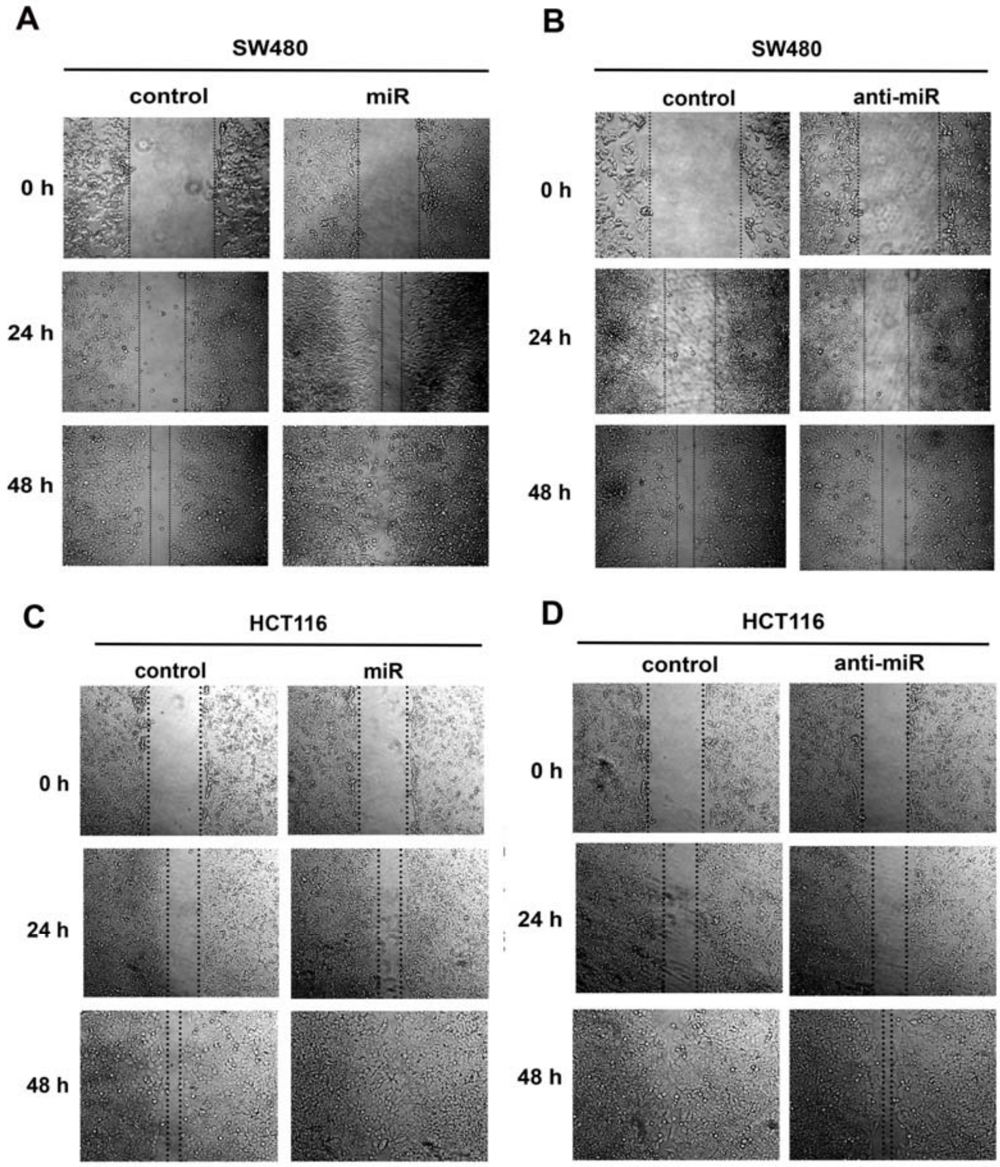

Supplementary Figure 1. Overexpression of miR-105 promoted CRC cells migration *in vitro*. (A) Representative images of wound-healing assay on control and miR-105 overexpressed SW480 cells. The distance migrated by treated cells was relative to that migrated by control cells. (B) Representative images of wound-healing assay on control and anti-miR-105 overexpressed SW480 cells. (C) Representative images of wound-healing assay on control and miR-105 overexpressed HCT116 cell lines. (D) Representative images of wound-healing assay on control and anti-miR-105 overexpressed HCT116 cell lines.

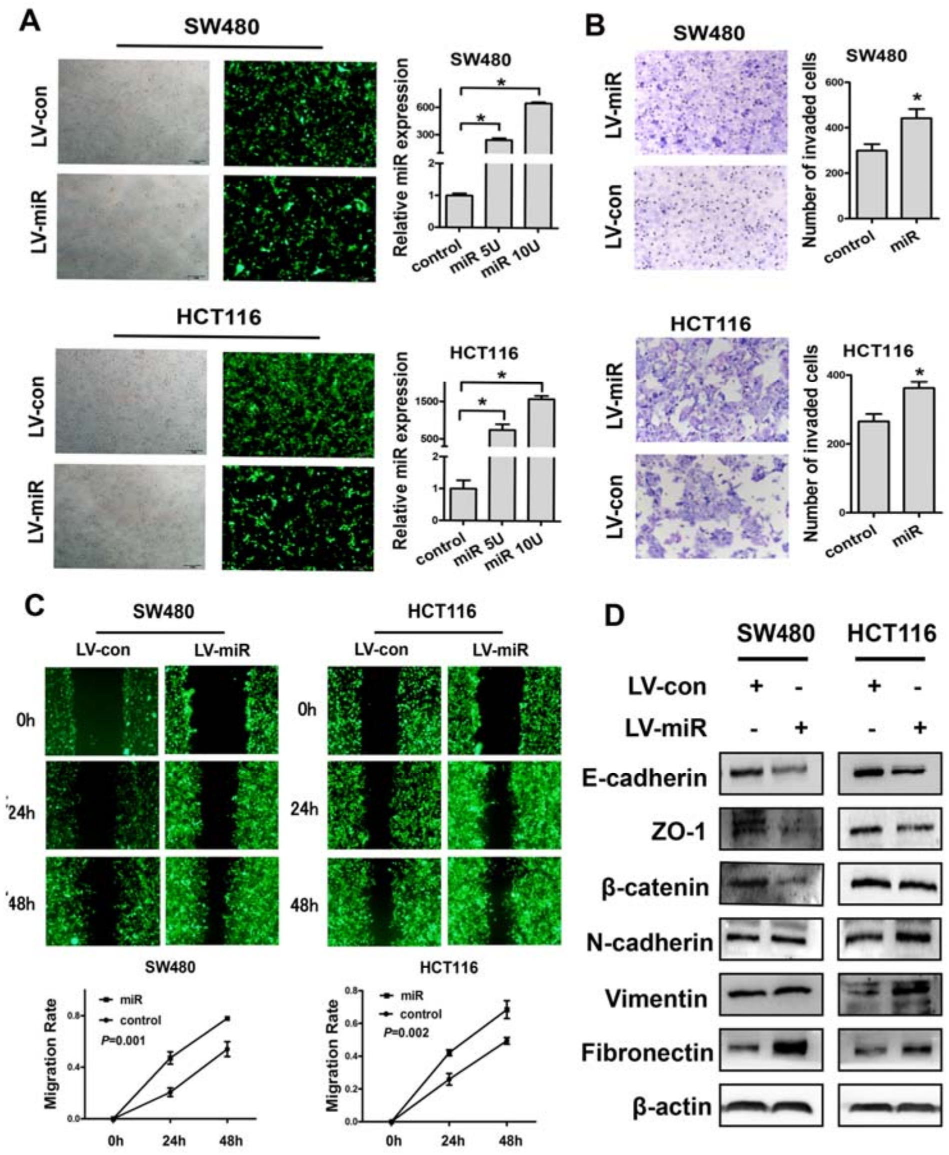

Supplementary Figure 2. Stable overexpression of miR-105 using Lenti-virus system promoted aggressive phenotypes of CRC cells *in vitro*. (A) The expression of miR-105 was remarkably increased in both LV-miR-105 infected SW480 and HCT116 cells compared with LV-miR-NC infected cells ($P < 0.05$). Intensity of green fluorescence indicates the expression level of miR-105. Bars on the right represent the relative miR-105 expression level after normalization to the expression of U6. (B) MiR-105 stable overexpressing SW480 and HCT116 cells obviously enhanced migration ability using transwell assay ($P < 0.05$). (C) MiR-105 stable overexpressing SW480 and HCT116 cells obviously enhanced migration ability using wound-healing assay($P < 0.05$). (C) Expression of the epithelial markers and the mesenchymal markers in control and miR-105 transfected SW480 and HCT116 cells were assessed by western blot. β-actin was used as a loading control.

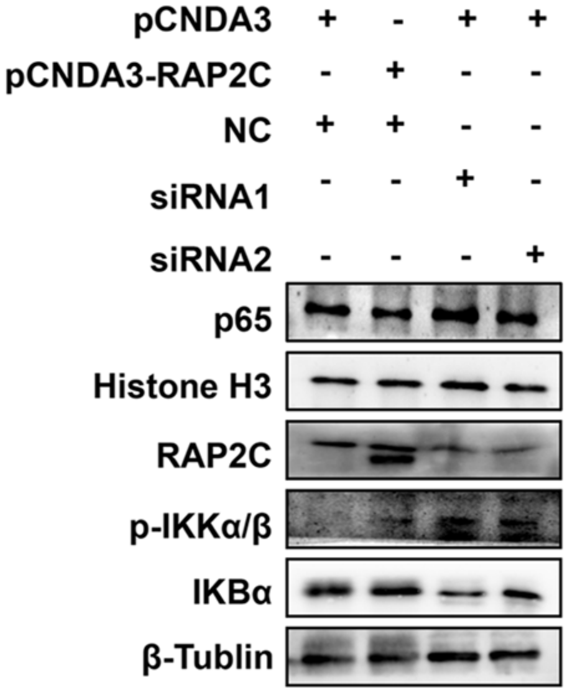

Supplementary Figure 3. RAP2C plays crucial roles in TNF-α/miR-105/NF-κB-induced EMT of CRC cells. Western blotting analysis detected the expression of NF-κB family members in HCT116 cells in response to RAP2C as well as silencing of RAP2C.

Supplementary Table 1. Primers used in Real-time qPCR

Gene/miRNA Name	Sequences
miR-105-5p	GGAGCGAGATCCCTCCAAAAT
GAPDH	GGAGCGAGATCCCTCCAAAAT<br>GGCTGTTGTCATACTTCTCATGG
U6	ATGGACTATCATATGCTTACCGTA<br>TACGGTAAGCATATGATAGTCCAT
MCC	AATAAACGTCTCCAGCAAACAGA<br>CGTTCCTCATAGCGAAGTGTC
PRDM2	TTTCACTACCGCCATTCGCT<br>CCACGCCATTTTTGGGTTGC
AMOT	AGGCAAGAGTTGGAAGGATGC<br>AGGATGACTTCACGAGGTTCT
CAMTA1	GAAGAGCGTTTCCCAAAGTG<br>ATTTCGGCAGACATTCAAGC
ZBTB11	GGCGGGACTCTGTATTACCA<br>TCCTGGCATTTGCTACACTG
MIER1	AGGCTAATAAAGTCCGAACAAGG<br>TTCTTTCCAAATCGTGTTTGCTG
USP2	GCCCTCGTGGAAGAGTTTGC<br>CTCGGTTCACCTCGTTATGG
NEDD4L	GGAATTGATCTCGCCAAAAA<br>CAGGAAGTCGTCTCGTGTCA
PGRMC2	ATGGGAAAGTCTTCGACGTG<br>AACTGCATTTCCCATTCTCG
FUT9	CCATTTGGGCAGACCTTTGAC<br>AGAACTGCATGGGATTTGTTGT
RAP2C	ACCTCAATCATGGCCATACC<br>GATGGATCTTGAAGGCCAAA
PTPRG	ACCCGTACTGGGCCTACTCT<br>CACGCGCATACTGGTCTAAA
RB1CC1	GAAAGAGCTTGCTCAGGGATT<br>TCATCAACTGATTTGCGTGACT
USP46	AGAAGAAGGTTGGCGTCATCC<br>TGTCCGCAATAGTGTTTAGCAAA
FLRT2	ACGGCTGATAACTTGCCATC<br>CAGCTAGAGGGAACGTCCTG
RAB2A	CTTTTGCACGAGAACATGGA<br>GCATGTGTTGCATTGGTAGC
EPHA4	TACCCCGCGAATGAAGTTAC<br>GTTATTCTGGCTGGGTTCCA
SEL1L	AAACCAGCTTTGACCGCCAT<br>GTCATAGGTTGTAGCACACCAC
ARHGEF7	TGCTTTCAACGTACCTACGGC

	GGCAACTTGGTGCATTCTTCTAA
RERG	ACCTACCGACACCAAGCAAC<br>CCCCTCCCTCTGAATGGTAT
REST	GCAACATTGGAATGGCCCTG<br>GTTATCCCCAACCGGCATCA
EIF4A2	GGTCAGGGTCAAGTCGTGTT<br>ACATTCATGGGCATCTCCTC
RAPGEF2	ATGAAACCACTAGCAATCCCAG<br>TGGGTGGAGACTGTCAGTAAG
LMO7	GTCTACAGTTCCGTCAAGAAGG<br>TCTGAAGGATAAGTTGCTCCCT
EXTL2	ACTGGAAACCAATGCAGTGTT<br>AGTAGAGACGTGCTTTCTAGGAA
AP3M1	CACCTGTCATTTCAACACCTCA<br>TCAGCAACTCGATGTAGGAACT
MTCH2	TGGCCTACCTCGTCAATACC<br>CACCAGCAAGACCACAGTTG
CERS6	GCAGGGATCTTAGCCTGGTTC<br>AAAAGCGAGATAGAGGTCCTCA
